# Tongue Image Texture Classification Based on Image Inpainting and Convolutional Neural Network

**DOI:** 10.1155/2022/6066640

**Published:** 2022-12-15

**Authors:** Jianjun Yan, Bochang Chen, Rui Guo, Menghao Zeng, Haixia Yan, Zhaoxia Xu, Yiqin Wang

**Affiliations:** ^1^Shanghai Key Laboratory of Intelligent Sensing and Detection Technology, East China University of Science and Technology, Shanghai 200237, China; ^2^Institute of Intelligent Perception and Diagnosis, School of Mechanical and Power Engineering, East China University of Science and Technology, Shanghai 200237, China; ^3^Comprehensive Laboratory of Four Diagnostic Methods, Shanghai University of Traditional Chinese Medicine, Shanghai 201203, China

## Abstract

Tongue texture analysis is of importance to inspection diagnosis in traditional Chinese medicine (TCM), which has great application and irreplaceable value. The tough and tender classification for tongue image relies mainly on image texture of tongue body. However, texture discontinuity adversely affects the classification of the tough and tender tongue classification. In order to promote the accuracy and robustness of tongue texture analysis, a novel tongue image texture classification method based on image inpainting and convolutional neural network is proposed. Firstly, Gaussian mixture model is applied to separate the tongue coating and body. In order to exclude the interference of tongue coating on tough and tender tongue classification, a tongue body image inpainting model is built based on generative image inpainting with contextual attention to realize the inpainting of the tongue body image to ensure the continuity of texture and color change of tongue body image. Finally, the classification model of the tough and tender tongue inpainting image based on ResNet101 residual network is used to train and test. The experimental results show that the proposed method achieves better classification results compared with the existing methods of texture classification of tongue image and provides a new idea for tough and tender tongue classification.

## 1. Introduction

Tongue diagnosis is one of the important diagnostic tools in traditional Chinese medicine and has great clinical value [1]. The tough tongue has a rough texture and is firm, which is the main evidence of actuality; the tender tongue has a delicate texture and is puffy and delicate, which is the main evidence of deficiency [2]. However, nowadays, the clinical diagnosis of tough and tender tongue mainly relies on the physician's visual observation and subjective judgment, and there is no objective judgment standard. Therefore, it is necessary to apply modern computer technology to research the classification method of tongue image texture and to realize the objectivity of the classification of tough and tender tongue.

Some scholars have already conducted research on the classification of tongue image texture. Xu et al. used grayscale difference statistics to describe the texture features of the tongue and analyzed the different trends of the four texture parameters in the tough, normal, and tender tongues to recognize tough and tender tongue [3]. Then, Liu et al. provided an algorithm to get the subimages of the tongue to which gray-level cooccurrence matrix (GLCM) was applied to extract the tongue texture features [4]. The subimages were classified by using these features. However, the characteristics of tough and tender tongue essentially contain two parts: color features and texture features. Only extracting the values describing texture features as the basis of classification makes the description of the characteristics of tough and tender tongue not comprehensive enough, which may affect the classification effect. Meanwhile, extracting the texture features of the tongue image as a whole as the basis of discriminating the characteristics of tough and tender tongue only achieves the classification of tough and tender tongue, ignoring the case of normal tongue. Cao et al. implemented the extraction of tongue color and texture fusion features and classification based on the k-nearest neighbor (KNN) classifier AdaBoost algorithm to build fusion features of tough and tender tongue for classification [5]. This method addressed the dilemma of lacking tongue color features, but it ignored the disturbing of tongue coat which may influence the accuracy of classification since the tongue image contains both tongue coating and body.

With the continuous development of convolutional neural networks, deep learning is increasingly used in the field of image classification. By simulating the structure of the human nervous system, convolutional neural networks pass information layer by layer and automatically extract the corresponding features, which can more fully and completely extract the color and texture information in images and achieve better classification results compared with specific color and texture feature description methods. Using convolutional neural networks to classify tough and tender tongue images can overcome the problems of instability and susceptibility to different light sources of traditional texture feature extraction methods [6]. However, this method is susceptible to the interference of tongue coating in tongue images, which affects the accuracy of tough and tender tongue classification.

Therefore, this paper proposes an image inpainting and convolutional neural network-based tongue image texture classification method to complete the classification of three different types of tongue body texture: tough tongue, tender tongue, and normal tongue. The rest of this article can be divided into three parts.  Section 2 described overall process of the tongue image texture classification.  Section 3 described the details of the experiment and discussed the results. Finally, the conclusion was drawn in Section 4.

## 2. Research Methodology

### 2.1. System Overview

**Figure 1:**
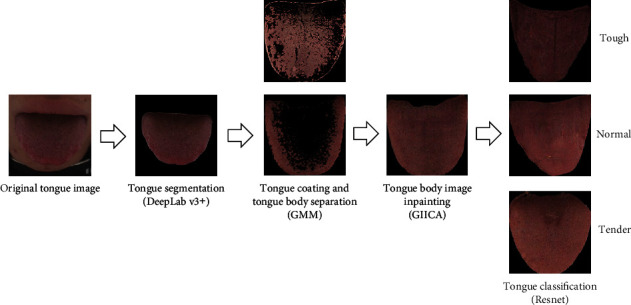
Flowchart of tough and tender tongue identification.

The overall process of the proposed tough and tender tongue classification method is shown in Figure 1. Firstly, the tongue image was segmented by DeepLab v3+ [7, 8] semantic segmentation model. Secondly, since tough and tender tongue is mainly related to tongue texture, the Gaussian mixture model (GMM) clustering was used to separate tongue coating from the tongue image, and the image containing only tongue body was obtained. The tongue texture in the obtained tongue body image is discontinuously distributed in various parts of the tongue body, and texture discontinuity will interfere with the classification of tough and tender tongue. Therefore, this paper used generative image inpainting with contextual attention (GIICA) [9, 10] network to realize the inpainting of the tongue body image, so as to eliminate the interference of the tongue coating on the classification of the tough and tender tongue images and obtain the tongue body image with continuous texture features and color changes. Finally, a convolutional neural network was employed to construct the classification model of tough and tender tongue images.

### 2.2. Tongue Image Segmentation

The backgrounds of acquired raw tongue image such as lips and surrounding skin in addition to the tongue body can interfere with the classification of the tough and tender tongue. Therefore, tongue image segmentation is needed for the raw tongue image. In this paper, a semantic segmentation model of tongue body was trained based on DeepLab v3+ semantic segmentation network. DeepLab v3 obtains the prediction result directly after acquiring contextual information through ASPP module, but it cannot obtain clear boundary information by using this module alone, so encoder-decoder architecture is added in DeepLab v3+, and the encoder module in this architecture is able to perform semantic extraction with ASPP to obtain richer information, while the decoder module is used to recover the boundary information. Combining the encoder-decoder architecture with the ASPP module results in the DeepLab v3+ architecture, which has become a widely used deep learning semantic segmentation method in medicine today. The tongue contours segmented using the DeepLab v3+ tongue semantic segmentation model are clear and accurate, and the lips, skin, and other backgrounds are well removed, facilitating subsequent tongue image feature analysis and classification.

### 2.3. GMM-Based Tongue Coating and Body Separation

**Figure 2:**
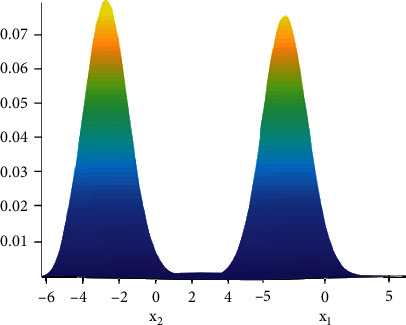
Schematic diagram of 2D Gaussian mixture model.

Among the characteristics of tough and tender tongues, tough tongues have rough texture with firm and tough shape, while tender tongues have fine texture with puffy and delicate shape. Therefore, in this paper, Gaussian mixture model clustering algorithm was used to separate the tongue coating from the tongue body, to get the tongue body image, to exclude the interference of tongue coating on the classification of tongue body, and to prepare for the subsequent tongue body image inpainting. GMM refers to the linear combination of multiple Gaussian distribution functions (normal distribution), and it is also the fastest learning probability model; GMM tries to find the mixture of multidimensional Gaussian distributions that can best simulate the input dataset [11, 12]. Its schematic diagram is shown in Figure 2.

Assuming the existence of *d* dimensional random variables *x* = (*x*^1^, *x*^2^, ⋯⋯,*x*^*w*^)^*T*^, the Gaussian mixture model with *K* components can be expressed as Equation (1) [11, 13].

(1) $$\begin{array}{c} \left\{\begin{array}{c} p\left(x\right)=\overset{K}{\underset{k=1}{\sum }}\omega_{k}N\left(x\;|\;\mu_{k},\sum k\right)\\ \left(x\;|\;\mu_{k},\sum k\right)=\frac{1}{\sqrt{\left|\sum k\right|}{\left(2\pi \right)}^{d/2}}\exp\left\{-\frac{1}{2}{\left(x-\mu_{k}\right)}^{T}\sum k^{-1}\left(x-\mu_{k}\right)\right\}\\ \overset{K}{\underset{k=1}{\sum }}\omega_{k}=1,0\leq \omega_{k}\leq 1 \end{array}\right., \end{array}$$

where *N*(*x* | *μ*_*k*_, ∑*k*) is the Gaussian probability density function; *ω*_*k*_, *μ*_*k*_, and ∑*k* are the weights, means, and covariance matrices of the *k* component of the GMM, T means matrix transpose.

It has been proven that many datasets conform to Gaussian distribution, even if the original dataset does not conform to Gaussian distribution, but as the number of samples increases, it will converge to Gaussian distribution according to the central limit theorem. And theoretically, by increasing the number of models, GMM can converge to any continuous probability density distribution, which makes it suitable for more flexible class cluster shapes [14–16]. Compared to K-means, GMM has higher computation time than K-means. Another limitation is that GMM uses all the components it has access to, which may add difficulty to initialization of clusters when data dimensionality is high. Despite that, in the process of clustering, GMM clustering output after training is not a specific value like K-means clustering but a set of probability values (probability of belonging to different classes). It is more accurate to determine which category the data belongs to by the magnitude of the probability values belonging to different categories than to directly assign the data to a certain category, especially when there is an overlap between different categories of clustering; it is very easy to confuse the category to which the data belongs directly, so the accuracy of GMM clustering is higher than that of K-means clustering [11]. GMM clustering has a better effect on the separation of tongue body and coating, which is more accurate and can better exclude the interference of tongue coating on the classification of tough and tender tongue and is also beneficial to the subsequent inpainting of tongue body images.

### 2.4. GIICA-Based Tongue Image Inpainting

In order to maintain the continuity of tongue texture and color changes while excluding the interference of tongue coating in the raw tongue image on the classification of tough and tender tongue features, so that the cluttered texture and color changes do not interfere with the classification of tough and tender tongue, the inpainting of tongue body image is needed. Since tough and tender tongue is closely related to tongue texture features and traditional image inpainting algorithms are less effective in to realizing the inpainting of large broken areas and cannot recover detailed textures better, current deep learning-based image inpainting methods have achieved better results for the challenging task of restoring a large number of missing areas in images.

Although the general deep learning-based image inpainting methods can generate visually reasonable image structures and textures, the restored regions usually produce distorted structures or blurred textures that are inconsistent with the surrounding regions because the convolutional neural networks cannot explicitly replicate and utilize the textures from the distant space. To address this problem, Yu et al. [17] proposed a generative image inpainting with contextual attention network based on a content attention mechanism. The network not only synthesizes new image structures but also explicitly uses the surrounding image features as references during network training in order to make better predictions and has better results for the repair of large defective regions of images, and the repair model established by this network can be used for the repair of any shape-deficient regions of arbitrary resolution images. Therefore, in this paper, the GIICA network was used to establish a tongue image inpainting model to achieve the inpainting of tongue images.

**Figure 3:**
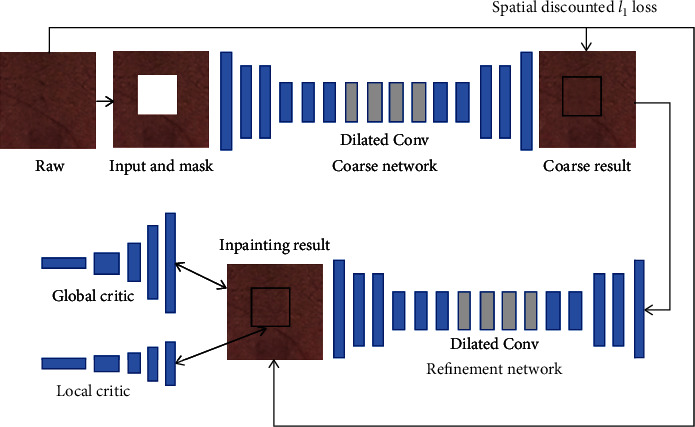
Schematic diagram of coarse-to-fine network architecture.

The GIICA network first constructs its base generative image inpainting network by replicating and improving the globally and locally consistent image completion (GLCIC) algorithm [18], and then introduces a rough-to-fine network architecture, in which the first network makes initial rough predictions of the missing region, and the second network takes the rough prediction result as input and makes precise prediction to finally complete the inpainting of the missing region. This architecture is also known as coarse-to-fine network architecture which is shown in Figure 3.

**Figure 4:**
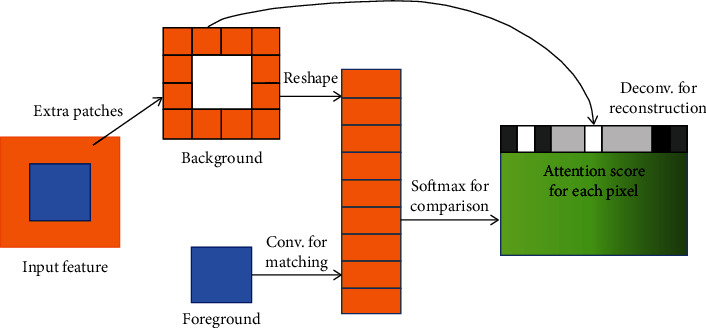
Schematic diagram of content-aware layer.

In addition, since convolutional neural networks process features of image with local convolutional kernels layer by layer, they are less capable of extracting features from the location far away from the missing region. Therefore, in the next part of the GIICA network, in order to overcome this limitation, the perception mechanism is considered, and a new contextual attention layer (CAL) is introduced in the deep generative network. CAL is used to learn to generate the missing patches by drawing feature information from somewhere in the known region of the image. Since CAL is differentiable, it can be trained in deep models and full convolutional networks and can be tested on images of arbitrary resolution. CAL works as shown in Figure 4.

From Figure 4, it can be seen that CAL uses convolution to calculate the matching scores of foreground patches and background patches, and then applies softmax to compare and obtain the attention score of each background pixel, and finally reconstructs the foreground patches with background patches by deconvolution of the attention score of each background pixel, so as to overcome the drawback that general convolutional neural networks cannot make use of the distant background information.

To integrate the improved generative image inpainting network with the content-aware module, the GIICA network introduces two parallel encoders based on the rough prediction results of the first encoder-decoder. The bottom encoder in Figure 3 is dedicated to the imaginary content of the layer-by-layer convolution, while the top encoder tries to participate in the content-aware function, and finally, the output features from both encoders are aggregated and fed to a single decoder to obtain the final inpainting results.

**Figure 5:**
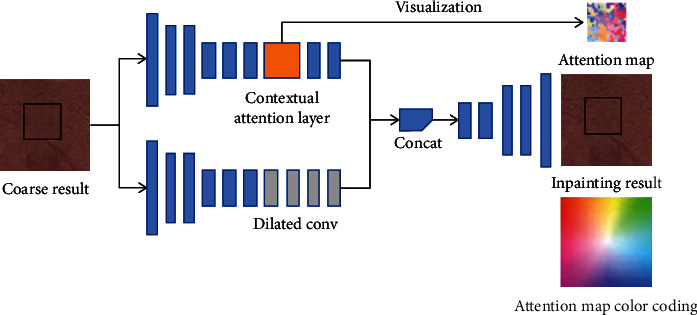
Schematic diagram of parallel encoder fusion based on rough prediction results.

Figure 5 visualizes the attention map, where the colors in the map represent the relative positions of the most interested background color blocks corresponding to each pixel in the foreground. The position of each color in the attention map color coding represents the relative position of the most interesting background color block of each pixel in the attention map, for example, white indicates that the most interesting region is on itself, pink indicates that the most interesting background color block of the corresponding foreground region is on the lower left, and green indicates that it is on the upper right.

### 2.5. ResNet-Based Tough and Tender Tongue Image Classification

After all the image processing, ResNet is used to train and test the classification model of tough and tender tongue inpainting image. Convolutional neural network (CNN) [19] is a class of deep neural networks containing convolutional computation, which is widely used in image classification. The ResNet draws on the advantages of traditional deep learning networks and introduces the residual learning method, which solves problems such as loss and loss of information in transmission; makes the whole network only need to learn the difference between input and output, simplifying the goal and difficulty of network learning; effectively solves the problem of gradient dissipation and gradient explosion that exists in deep networks, enabling the network to be as deep as possible [20, 21].

**Figure 6:**
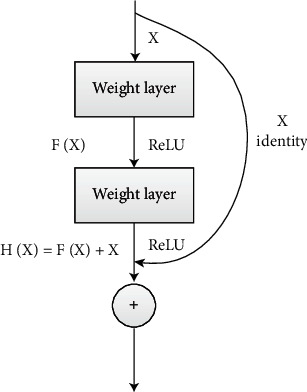
Residual block diagram.

ResNet introduces constant mapping and residual mapping based on the traditional convolutional neural network.  Figure 6 shows a typical residual block, and the constant mapping is the process shown in the curve (short connection) in Figure 6, which is a mapping that can skip 2 layers of weight layers (the number of layers is variable, it can be 3 or 4 layers) and then send *X* to the ReLU layer. Since *X* skips directly over the weight layers without any operation, it is called a constant mapping, i.e., *G*(*X*) = *X*. The linear process represents the residual mapping, which is the difference between the input and the output, i.e., *F*(*X*) = *H*(*X*) − *X*.

This simple operation does not increase the parameters and computation of the network but can greatly increase the training speed of the model and improve the training effect. Moreover, due to the identity map, the gradient can be directly returned to the shallow layer when back propagating, which effectively solves the problem of model degradation when the network is deepened. The ResNet is formed by applying the residual blocks several times on the basis of the traditional convolutional neural network, which makes it possible to train extremely deep learning neural networks. The most common deep residual networks are mainly ResNet50 and ResNet101. The performance comparison of ResNet50 and ResNet101 on ImageNet validation dataset is shown in Table 1 [19].

Table 1 shows that the Top-1 error rate and Top-5 error rate of ResNet101 on ImageNet validation dataset are 0.87% and 0.65%, which are, respectively, lower than those of ResNet50. It can be seen that ResNet101 has better image classification results on the ImageNet validation dataset compared to ResNet50. Therefore, ResNet101 is selected in this research to build the tough and tender tongue classification model.

**Figure 7:**
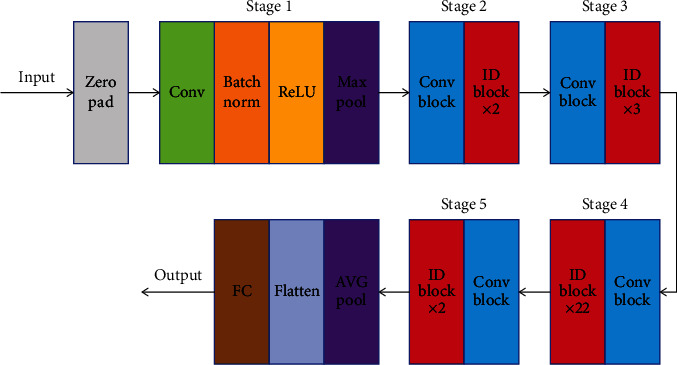
ResNet101 structure diagram.

ResNet101 is a residual network with a 101-layer network structure, including 100 convolutional layers and 1 fully connected layer, and its specific structure is shown in Figure 7:

Zero padding means zero complement to input, CONV in stage1 means one layer of convolution, and Batch Norm means batch normalization. During the process of training neural networks, the distribution of each layer's inputs is affected by the randomness of the parameter initialization and input data. The phenomenon is called internal covariate shift. So, low learning rates and careful parameter initialization are usually needed. But Batch Norm, which adds two parameters during normalization, allows us to use much higher learning rates and be less careful about initialization by reducing the internal covariate shift [22]. Meanwhile, Batch Norm has a regularizing effect such that the network improves its generalization properties; thus, it is unnecessary to use dropout to mitigate overfitting. The network becomes more robust to different initialization schemes and learning rates after Batch Norm. ReLU means an activation function called rectification linear units ranging from zero to infinity. It suffers from a restriction called dead ReLU problem; during training process, a part of neurons may die, which means the activation output is always zero and the corresponding parameters will not update anymore. A neuron dies when its weights get tweaked in such a way that the weighted sum of its inputs is negative for all instances in the training set. To avoid the problem, learning rates need to be set properly. MAX POOL means maximum pooling, CONV BLOCK in stage2-5 means residual block that changes the scale of feature vector, ID BLOCK × 2 means 2 residual blocks that do not change the scale of feature, ID BLOCK × 3 means 3 residual blocks that do not change the scale of feature (in ID BLOCK × *N*, ID BLOCK means residual block that does not change the scale of feature, and *N* means the number of the residual blocks), and each residual block includes 3 convolutional layers; from Figure 7, we can see that there are 1 + 3 × (3 + 4 + 23 + 3) = 100. After the feature operations of stage1-5, the resulting feature vector is AVG POOL (mean pooling), and then the multidimensional feature vector is flattened to obtain a one-dimensional feature vector, which is then fed into the fully connected layer (FC) to obtain the output, and finally, the softmax classifier calculates the probability of each category to get final classificaiton results of tough and tender tongue images.

## 3. Experiment

### 3.1. Experimental Data and Preprocessing

The tongue image dataset used in the experiment was obtained from the Comprehensive Laboratory of Four Diagnostic Methods of Shanghai University of Traditional Chinese Medicine, with a total of 953 tongue images, including 530 tough tongue images, 198 tender tongue images, and 225 normal tongue images. Meanwhile, there is a slight sample imbalance in the tough and tender tongue classification dataset, and tender and normal tongue images are less than tough tongue images, which will reduce the generalization ability of the model and make it easy to overfit if the model is trained with this dataset. Therefore, in this paper, horizontal mirroring and resizing were used to balance the dataset and obtain 700 images of tough tongue, tender tongue, and normal tongue, totally 2100 tongue images. The 600 images of each of the three types of tongue body were used as the training set, and the remaining 100 images were used as the test set.

**Figure 8:**
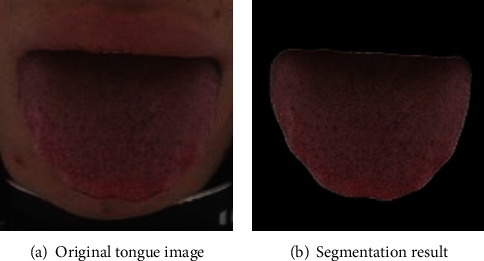
The effect of DeepLab v3+ semantic segmentation model.

Because the captured original tongue image contains backgrounds such as lips, skin, and the inner wall of the collector box, which will interfere with the subsequent classification of the tough and tender tongue, it is necessary to perform tongue body segmentation on the original tongue image to remove other backgrounds and obtain the tongue image. The automatic segmentation of the tongue body based on DeepLab v3+ was realized, and the segmentation effect is shown in Figure 8.

**Figure 9:**
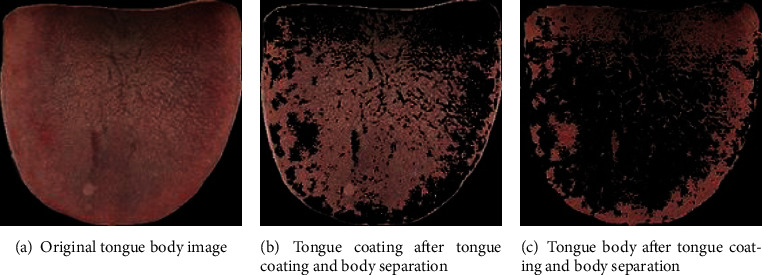
The effect of tongue coating and body separation.

In order to exclude the interference of tongue coating on the classification of tough and tender tongue features, GMM clustering method is utilized to achieve the separation of tongue coating from tongue images based on Visual Studio 2015 and OpenCV 4.0. The effect of tongue coating and body separation based on GMM clustering is shown in Figure 9.

**Figure 10:**
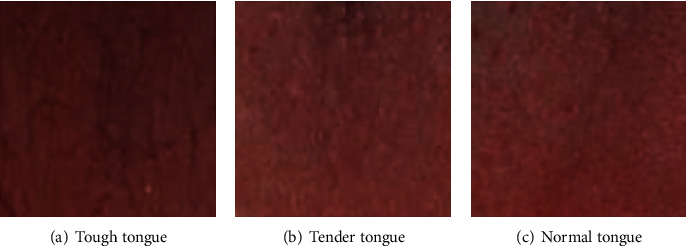
Small pieces of tongue body image.

Based on the deep learning framework Tensorflow, GIICA network is employed to establish a tongue image inpainting model to realize the inpainting of tongue images. Since the GIICA network training requires all tongue images as the dataset to build the tongue image inpainting model, and the tongue images basically contain both tongue coating and tongue body, which cannot be used as training data directly. Therefore, in this paper, after separating the tongue coating and body, the tongue body images would be traversed through *n* × *n* window to obtain small pieces of *n* × *n* pixels. Then, those small pieces with more than 80% of tongue body would be extracted as the training data needed to build the GIICA inpainting model. Large window size may lead to the lack of available small pieces of tongue body images, which will affect the training of tongue body image inpainting model. Therefore, after experiments, it is found that window size of 96 × 96 can make sure that most of the pieces contain enough tongue body pixels, so that enough data can be obtained to train and validate. Totally, 222 small pieces of tongue body images were obtained. The data set was expanded to 444 images by mirroring, of which 396 images were used as the training set, and 48 images were used as the validation set. The pieces with more than 80% of tongue body are shown in Figure 10.

**Figure 11:**
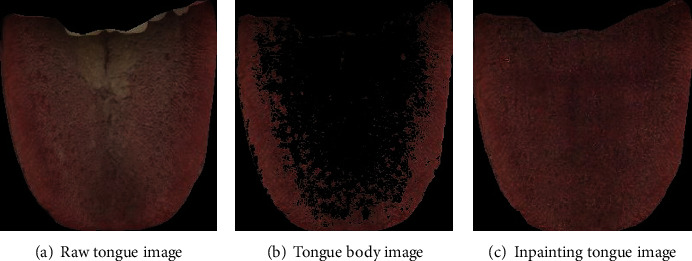
Inpainting effect of tongue body image.

The results of the tongue image inpainting is shown in Figure 11.

After the tongue image inpainting, the original tongue coating part was basically transformed to the tongue body, and the detailed texture of the inpainting area was well represented, which is conducive to the elimination of the interference of the tongue coating to the classification of the tough and tender tongue and the establishment of the subsequent classification model of the tough and tender tongue.

### 3.2. Experimental Results and Analysis

In this paper, the classification accuracy of the test set for classification of tough and tender tongue features is used as the experimental evaluation index, and its mathematical expression is as follows:

(2) $$\begin{array}{c} \text{accuracy}=\frac{R}{S}, \end{array}$$

where *R* denotes the number of correctly classified samples in the test set, and *S* denotes the total number of samples in the test set.

In this paper, we implemented ResNet101 deep residual network by the Keras framework and set the parameters as follows: the number of training epochs is 1000; initialized learning rate is 0.001. Batch size needs to be carefully set as it is closely linked to the accuracy of the estimation. Not only will large batch size occupy lots of memory, which will make GPU not work normally, but also will lead to local optimization while classification accuracy is low. Meanwhile, if batch size is too small, the greater randomness will make it more difficult to converge so that the learning speed will be slower. After some experiments, batch size is set to 10, which can make the learning process fast and accurate. The training step that needs to reduce the learning rate is 5 rounds, which means when val_loss does not decrease in 5 consecutive rounds, the learning rate reduces. The learning rate decay factor is 0.1, which means the learning rate decays at a rate of 10 times. The step to stop training early is 15 rounds which means when val_loss does not decrease in 15 consecutive rounds, the model training is terminated early. The software configuration of this experiment is Anaconda, Python 3.6, on which the deep learning environment Keras is built. The CPU in the hardware configuration is Intel Core i5-10300H, the memory is 16GB, and the GPU graphics card is NVIDIA GTX 1660Ti. Training the GIICA inpainting model of tongue body images and the classification model of tongue image texture based on ResNet will take 10 minutes and 40 minutes, respectively.

In order to verify the effectiveness of the ResNet101 residual network selected in this paper for tough and tender tongue classification, ResNet50 and ResNet101 classification models were established using the training set of tongue repair images, and the model classification effect was tested using the test set, and the test results are shown in Table 2.

As shown in Table 2, the classification accuracy of tough tongue, normal tongue, and tender tongue increased by 1.0%, 2.0%, and 2.0%, and the overall classification accuracy increased by 1.7% when compared with the model built by using ResNet50. Thus, it can be seen that the model built using ResNet101 has better classification results in the classification of tough and tender tongue.

**Figure 12:**
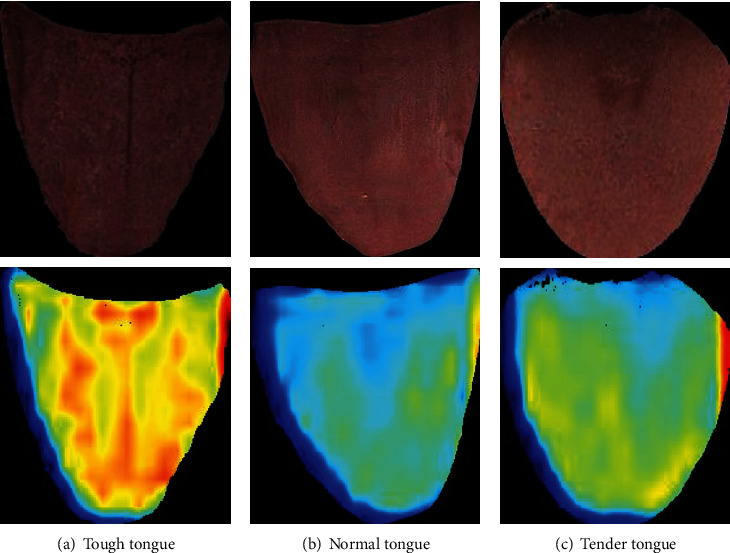
The examples of classification results.

One randomly selected tough tongue, one normal tongue image, and one tender tongue were each used as input to ResNet101 to obtain partial heat maps of the intermediate layers to analyze the effect of self-learning; the features of the three types of tongue repair images in the convolutional layer, and the original images and heat maps of the three types of tongue images are shown in Figure 12.

From Figure 12, it can be seen that the three types of tongue inpainting images, tough tongue, normal tongue, and tender tongue, are basically consistent with the color and texture descriptions of tough and tender tongue characteristics in TCM, with the inpainting tough tongue image having a rougher texture and duller color and the inpainting tender tongue image having a more delicate texture and slightly puffy shape. Most of the tough tongue images were orange-red in the thermogram, most of the normal tongue images were light green in the thermogram, and most of the tender tongue images were yellow-green in the thermogram. The heat maps of the three images reflect that there are obvious differences in the features of tough, normal, and tender tongues, which indicates that ResNet101 can learn the features of the three types of tongue images better and build a better classification model to identify tough and tender tongues.

The tongue body image with nonpretreatment and inpainting tongue image was input into the ResNet101 network separately to establish the tongue classification model, and the test results are shown in Table 3.

As can be seen from Table 3, the classification accuracy of ResNet101 models built from the dataset after preprocessing with GIICA was 83.0%, 98.0%, and 92.0% for tough, normal, and tender tongues, which was 5.0%, 6.0%, and 4.0% higher than the classification accuracy of the model built using the original tongue image training set, and the overall classification accuracy was 91.0%, an improvement of 5.0%.

The experiments illustrate that the tongue image inpainting based on GIICA network can effectively exclude the interference of tongue coating on tough and tender tongue classification. Using ResNet101 network to build a tough and tender tongue classification model can achieves better classification accuracy compared with the model built by using the raw tongue image as the training set.

In order to verify the effectiveness of the proposed tough and tender tongue classification method, the results of this paper were compared with the grayscale difference statistics (GDS) method [3] and the KNN-based AdaBoost algorithm [4]. The results are shown in Table 4.

As can be seen from Table 4, the classification method of this paper improved 23.0%, 26.0%, and 33.0% in the classification accuracy of tough tongue, normal tongue, and tender tongue and 27.3% in the overall classification accuracy compared with the grayscale difference statistics method; while compared with KNN+AdaBoost, the classification accuracy improved 14.0% and 22.0%, and the overall classification accuracy was improved by 20.0%. Thus, this paper used the GIICA tongue image inpainting model to obtain tongue images with continuous color and texture changes, and then used the ResNet101 network to self-learn the tongue inpainting image features, and the obtained features reflected the color and texture features of different tough and tender tongues more comprehensively than the tough and tender tongue features extracted manually. The final model of tongue body classification has achieved a satisfactory result.

In summary, this paper separated tongue coating based on GMM clustering algorithm for tough and tender tongue dataset, then obtained the inpainting tongue image by GIICA network, and then used the inpainting tongue image to build tough and tender tongue classification model with ResNet101, which has better classification effect compared with the model built by using tongue body image. The GIICA+ResNet101 method achieves a higher classification accuracy compared with the existing methods such as GDS and KNN+AdaBoost, which indicates that the proposed method achieves a more satisfactory accuracy effect in tough and tender tongue classification.

## 4. Conclusion

In this paper, an image inpainting and convolutional neural network-based tongue texture classification method is proposed. Firstly, GMM was used to separate the tongue body to eliminate the interference of tongue coating on the classification of tough and tender tongue; then, GIICA network was employed to establish a tongue image inpainting model to realize the inpainting of tongue image to eliminate the interference of discontinuous texture and color changes on the classification of tough and tender tongue; finally, a ResNet101 residual network was used to self-learn and establish a model for the classification of tough and tender tongue. The effect of different datasets and different methods to classify tough and tender tongue were compared and analyzed, and it was determined that the proposed method achieved better accuracy than the previous methods. This study provides a new way of thinking and a new method for tough and tender tongue classification. In this study, however, the precision and reliability of tongue coating and body separation based on GMM still need to improve. Also, small sample size and sample imbalance will influence the inpainting based on GIICA and the tongue texture classification based on ResNet. If the experimental samples can be enriched and the balance of samples can be improved, the accuracy for tongue image texture classification can be further improved.

## Figures and Tables

**Table 1:** Comparison of ResNet50 and ResNet101 performance (%).

	Top-1	Top-5
ResNet50	20.74	5.25
ResNet101	19.87	4.60

**Table 2:** Identification accuracy results of different networks (%).

	Tough tongue	Normal tongue	Tender tongue	Overall
ResNet50	82.0	96.0	90.0	89.3
ResNet101	83.0	98.0	92.0	91.0

**Table 3:** Classification accuracy results of different pretreatment methods (%).

	Tough tongue	Normal tongue	Tender tongue	Overall
Nonpretreatment	78.0	92.0	88.0	86.0
GIICA	83.0	98.0	92.0	91.0

**Table 4:** Classification accuracy results of different identification methods (%).

	Tough tongue	Normal tongue	Tender tongue	Overall
GIICA+ResNet101	83.0	98.0	92.0	91.0
GDS	60.0	72.0	59.0	63.7
KNN+AdaBoost	69.0	77.0	67.0	71.0

## Data Availability

The datasets generated and analyzed during the current study are not publicly available due to the confidentiality of the data but are available from the corresponding author on reasonable request.

## References

[1] Kirschbaum B. (2000). *Atlas of Chinese Tongue Diagnosis*.

[2] Xin Y., Guo X. Z. (2001). *Tongue Diagnosis: Traditional Chinese Medicine*.

[3] Jiatuo X., Yang S., Zhifeng Z., Changle Z., Yimin B., Wenshu L. (2003). Analysis and identification of tongue texture features based on differential statistical methods. *Journal of Shanghai University of Traditional Chinese Medicine*.

[4] Liu C. Y. (2006). *Research and Application of Tongue Image Classification Based on Texture Features, [Ph.D. thesis]*.

[5] Cao M. L., Zhang X. F., Shen L. S. (2006). Research on the application of classifier fusion technology in the recognition of tough and tender Chinese medicine tongue images. *Beijing Biomedical Engineering*.

[6] Zhang K. Y., Zhang X. F., Ahmad F. Tongue image texture classification based on Xception.

[7] Chen L. C., Papandreou G., Schroff F., Adam H. Rethinking atrous convolution for semantic image segmentation. https://arxiv.org/abs/1706.05587.

[8] Chen L. C., Zhu Y., Papandreou G., Schroff F., Adam H. Encoder-decoder with Atrous separable convolution for semantic image segmentation.

[9] Kayabol K., Kutluk S. (2016). Bayesian classification of hyperspectral images using spatially-varying Gaussian mixture model. *Digital Signal Processing*.

[10] Li X. L., Wu Y. B. (2014). Image object detection algorithm based on improved Gaussian mixture model. *Journal of Multimedia*.

[11] Xu L., Jordan M. I. (1996). On convergence properties of the EM algorithm for Gaussian mixtures. *Neural Computation*.

[12] Vanderplas J. (2016). *Python Data Science Handbook*.

[13] Li K., Ma Z., Robinson D., Ma J. (2018). Identification of typical building daily electricity usage profiles using Gaussian mixture model-based clustering and hierarchical clustering. *Applied Energy*.

[14] Yang C., Chen C., He W., Cui R., Li Z. (2019). Robot learning system based on adaptive neural control and dynamic movement primitives. *IEEE Transactions on Neural Networks and Learning Systems*.

[15] Calinon S., Guenter F., Billard A. (2007). On learning, representing, and generalizing a task in a humanoid robot. *IEEE Transactions on Systems, Man, and Cybernetics, Part B (Cybernetics)*.

[16] Patel E., Kushwaha D. S. (2020). Clustering cloud workloads: K-means vs Gaussian mixture model. *Procedia Computer Science*.

[17] Yu J., Lin Z., Yang J., Shen X., Lu X., Huang T. S. Generative image inpainting with contextual attention.

[18] Iizuka S., Simo-Serra E., Ishikawa H. (2017). Globally and locally consistent image completion. *ACM Transactions on Graphics*.

[19] Zhang X., Fu C., Zhao Y., Xu X. (2020). Hybrid feature CNN model for point cloud classification and segmentation. *IET Image Processing*.

[20] He K., Zhang X., Ren S., Sun J. Deep residual learning for image recognition.

[21] Alotaibi B., Alotaibi M. (2020). A hybrid deep ResNet and inception model for hyperspectral image classification. *PFG–Journal of Photogrammetry, Remote Sensing and Geoinformation Science*.

[22] Ioffe S., Szegedy C. Batch normalization: accelerating deep network training by reducing internal covariate shift.

